# Disability and disease-related damage in Thai children and adolescents with juvenile idiopathic arthritis

**DOI:** 10.1186/s12969-023-00852-5

**Published:** 2023-07-10

**Authors:** Sirikarn Tangcheewinsirikul, Maynart Sukharomana, Sirirat Charuvanij

**Affiliations:** Division of Rheumatology, Department of Paediatrics, Faculty of Medicine Siriraj Hospital, Mahidol University, 2 Wanglang Road, Bangkoknoi, Bangkok, 10700 Thailand

**Keywords:** Articular damage, Children, Damage, Disability, Juvenile idiopathic arthritis

## Abstract

**Background:**

Children and adolescents with juvenile idiopathic arthritis (JIA) may suffer from disability and disease-related damage. This study aimed to investigate the prevalence of disability and damage, and identify the factors associated with articular and extra-articular damage in children and adolescents with JIA in a resource-restricted setting in Thailand.

**Methods:**

This cross-sectional study enrolled JIA patients during June 2019-June 2021. Disability was assessed using the Child Health Assessment Questionnaire (CHAQ) and Steinbrocker classification criteria. Damage was evaluated using the Juvenile Arthritis Damage Index (JADI) and the modified-JADI (mJADI) tools.

**Results:**

There were 101 patients (50.5% female) with median age of 11.8 years. Median disease duration was 32.7 months. Enthesitis-related arthritis (ERA) was the most common subtype (33.7%), followed by systemic JIA (25.7%). Thirty-three (32.7%) patients had delayed diagnosis ≥ 6 months. Moderate to severe disability was found in 20 (19.8%) patients. Patients with Steinbrocker functional classification > class I were seen in 17.9%. Thirty-seven (36.6%) patients had articular damage. Extra-articular complications were observed in 24.8%. Growth failure and striae were the most common complications in 7.8%. Leg-length discrepancy was documented in 5.0%. Ocular damage was found in 1 patient with ERA. Multivariable logistic regression analysis revealed Steinbrocker functional classification > class I (aOR: 18.1, 95% CI: 3.9–84.6; *p* < 0.001), delayed diagnosis ≥ 6 months (aOR: 8.5, 95%CI: 2.7–27.0; *p* < 0.001), and ERA (aOR: 5.7, 95%CI: 1.8–18.3; *p* = 0.004) as independent predictors of articular damage. Systemic corticosteroids use was the independent predictor of extra-articular damage (aOR: 3.8, 95%CI: 1.3–11.1; *p* = 0.013).

**Conclusions:**

Disability and disease-related damage was identified in one-fifth and one-third of JIA patients. Early detection and treatment are essential for preventing permanent damage.

## Background

Juvenile idiopathic arthritis (JIA) is the most prevalent etiology of childhood-onset chronic arthritis [1]. The aims of treatment are to reduce inflammation and to prevent complications and long-term disability. The therapeutic approaches are based on the recommendations of professional organizations established in developed countries [2–4]. However, resource-restricted countries may have limited access to biologic agents and an inadequacy of paediatric rheumatologists [5, 6]. As such, children with JIA in countries lacking these resources could develop further disease damage and experience an overall poorer outcome.

Disability and articular damage in patients with JIA have been variously reported [7–12]. Disabilities in JIA patients adversely affect their ability to perform activities of daily living and their overall quality of life [13, 14]. Physical disability was reported to exert the most pronounced adverse effect on quality of life in children with JIA [14]. Systemic JIA was reported to result in the most disabilities [7–9]. Polyarticular JIA and enthesitis-related arthritis (ERA) were associated with articular damage [7, 11, 12]. Growth restriction and pubertal delay were found to be issues of concern in children with JIA [7, 8]. Moreover, complications resulting from pharmacologic treatment including transaminitis, cataract, and low bone mass were demonstrated in patients with JIA [15, 16]. Long disease duration was significantly associated with disease-related damage [9, 12, 17].

Data specific to the prevalence of disability and disease-related damage in children and adolescents with JIA in resource-restricted Southeast Asia countries, like Thailand, are scarce. Accordingly, the aim of this study was to investigate the prevalence of disability and disease-related damage, and to identify the factors associated with damage in Thai children and adolescents with JIA.

## Methods

This cross-sectional study enrolled participants during June 2019 to June 2021 at the Paediatric Rheumatology Clinic of the Division of Rheumatology, Department of Paediatrics, Faculty of Medicine Siriraj Hospital, Mahidol University, Bangkok, Thailand. With 2,200 beds, Siriraj Hospital is Thailand’s largest university-based tertiary referral centre. There are 303 beds for paediatric in-patients’ admissions. Regarding Paediatric Rheumatology service, there are approximately 1,200 out-patient visits/year with currently 2 full-time paediatric rheumatologists. Eligible criteria for participation in this study included patients who were diagnosed with JIA according to ILAR classification criteria and had been followed-up for at least 6 months. [1] Assent or written informed consent was obtained from children, adolescents, and parents by research assistance who was not involved in patient care. The study protocol was approved by the Ethics Committee for Research in Humans of the Siriraj Institutional Review Board (SIRB) (COA no. Si 432/2019).

The following patient data were collected at the study visit: age, sex, weight, height, body mass index (BMI), pubertal stage, JIA subtype, disease activity status, and current medications used. Age at first symptom onset, age at JIA diagnosis, health benefit, previous treatments and comorbidities were retrospectively reviewed from the electronic medical record.

JIA disease activity assessment included active joint count, limited joint count, enthesitis count, modified Schöber’s test, physician global assessment of overall disease activity (PGA), parent’s or patient’s assessment of overall well-being (PGW), erythrocyte sedimentation rate (ESR), and C-reactive protein (CRP) level. The follow-up schedules were every 1–3 months depending on disease severity, in which clinical assessments, laboratory assessments, and anthropometric measurements were performed in every clinic visit. Disease activity was evaluated using the Juvenile Arthritis Disease Activity Score (JADAS)-71 assessment tool [18]. The JADAS-71 was evaluated in every clinic visit to determine the JIA disease activity. The Wallace criteria were used to define inactive disease [19].

Disability was evaluated using the Thai version of the Childhood Health Assessment Questionnaire (CHAQ) [13]. The CHAQ is a questionnaire that assesses a patient’s functional ability in 8 domains, including dressing and grooming, arising, eating, walking, hygiene, reaching, gripping, and activities. Scoring for each line item ranges from 0 to 3, with a 0 indicating ‘without any difficulty’, and a 3 indicating ‘unable to do’. The highest score given for any line item within a domain determines the overall score for that domain [20]. A patient with a CHAQ score of 0.6–1.5 was defined as moderate disability and the CHAQ score > 1.5 was regarded as having severe disability [8, 21].

Physical function was evaluated using the Steinbrocker functional classification, comprises the four following classes: (I) complete functional capacity, ability to perform all usual duties; (II) ability to conduct normal activities with limited mobility; (III) moderate restriction, inability to complete most duties of usual occupation or self-care; and, (IV) incapacitation or confinement to wheelchair, allowing little or no self-care [22].

The Juvenile Arthritis Damage Index (JADI) was used to assess articular damage (JADI-A), and the JADI-E was used to assess extra-articular damage [23]. The modified JADI (mJADI) scoring tools were developed to evaluate articular (mJADI-A) and extra-articular damage (mJADI-E) in patients with ERA [24]. JADI-A and mJADI-A scoring covers irreversible articular damage at both sides of all joints, except for the temporomandibular joint, cervical spine, tarsal bone, and lumbar spine, which were counted as one. The damage observed at each joint is scored on a 2-point scale, with a score of 1 given for partial damage, and a score of 2 given for severe damage, ankylosis, or prosthesis. Joint deformities and contractures in our study were scored only if they had been attributed to the past damage, not caused by currently active arthritis, and persisted for at least 6 months [23]. The total score for the JADI-A in non-ERA patients is 72 [23], and the total score for the mJADI-A in ERA patients is 78 [24]. The maximal JADI-E score in non-ERA patients is 17 [23], and the maximal mJADI-E score in ERA patients is 18 [24]. Articular and extra-articular damage were defined by a JADI-A or mJADI-A and JADI-E or mJADI-E score ≥ 1. The disability and damage were evaluated and collected at the last follow up visit.

The treatment primarily followed the recommendations of professional organizations [2–4, 25, 26]. For oligoarticular JIA, the initial treatment was NSAID and/or intraarticular corticosteroids injection. For polyarticular JIA and ERA, initial treatment included NSAID and DMARDs; methotrexate or sulfasalazine. Prednisolone was prescribed as bridging therapy in patients with moderate to high disease activity. For systemic JIA, initial treatment included NSAID and systemic corticosteroids. Intra-articular corticosteroids injections were mainly performed in oligoarticular JIA and as adjunctive treatments in other JIA subtypes [27]. Biologics treatment was indicated in JIA patients with refractory disease despite the combination of NSAID, DMARDs and corticosteroids.

All data analyses were performed using SPSS Statistics version 18.0 (SPSS, Inc., Chicago, IL, USA). The sample size was initially calculated by using the study reported by Menon et al. [9] by the formula: n = Z^2^_α/2_P(1-P)/d^2^ (α = type I error = 0.05, 2-sided, 95% CI, Z = 1.96; P = 0.301; d = 0.1), resulting in calculated sample size for at least 81.

Descriptive statistics were used to summarize demographic and clinical data. Comparisons of categorical data were performed using either chi-square test or Fisher’s exact test, and the results of those comparisons are given as number and percentage. Comparisons of continuous data were performed using either Student’s t-test (for normally distributed data) or Mann-Whitney U test (for non-normally distributed data). Data normality was evaluated by the Kolmogorov-Smirnov test. The results of continuous data comparisons are shown as mean ± standard deviation for normally distributed data, and as median and interquartile range [IQR] for non-normally distributed data. A logistic regression model was used to identify variables significantly and independently associated with articular damage. To control for potential confounding, variables with *p*-value in univariable analysis less than 0.1 were used for each variable of interest. The results of multivariable logistic regression analysis, which are given as adjusted odds ratio (aOR) and 95% confidence interval (CI), were calculated by enter method in binary logistic regression. A *p*-value of less than 0.05 was considered significant for all tests. We followed the reporting guideline of Strengthening the Reporting of Observational Studies in Epidemiology (STROBE) [28].

## Results

A total of 102 Thai children and adolescents were enrolled in this study. One of them was excluded due to the data of onset of symptom was missing. Of 101 remaining patients, 50 (49.5%) patients were female. The median [interquartile range, IQR] age at disease onset was 7.9 years [IQR: 3.8–9.9]. The median age at study visit was 11.8 years [IQR: 8.4–14.3]. The median follow-up duration was 32.7 months [IQR: 11.7–58.4]. The median duration [IQR] from the onset of symptoms to the time of assessment was 39.6 [17.9–65.4] months. The median time from symptoms onset to diagnosis was 3.0 months [IQR: 1.2–8.2]. Delayed diagnosis of ≥ 6 months was found in 33 (32.7%) patients. In those patients with delayed diagnosis of 6 months or more, the median (IQR) duration from the onset of symptoms to diagnosis was 13.0 [8.2–36.4] months.

ERA was the most common subtype, followed by systemic JIA, oligoarthritis, polyarthritis rheumatoid factor (RF) positive, polyarthritis RF negative, and undifferentiated JIA. One patient with RF + polyarticular JIA born from a mother who was diagnosed with RF + polyarticular JIA when she was young. The other adolescent ERA patient had mother diagnosed with ERA; both of them had + HLA-B27. No consanguinity was observed in our study cohort.

In Thailand, there are four main health care coverage schemes, including Universal Coverage scheme, Civil Servant Medical Benefit, Social Security scheme, and private health insurance or self-payment. The majority of patients in our study had the healthcare coverage through the Universal Coverage scheme supported by the Ministry of Public Health (77, 76.2%), followed by the Civil Servant Medical Benefit that the government provided support for government officers and their first-degree relatives (12, 11.9%) and self-payment (12, 11.9%).

Regarding JIA disease status, inactive disease was found in 34 (33.7%) patients. Remission on medication and remission off medication were presented in 4 (4%) and 28 (27.7%) patients, respectively. The median JADAS-71 score was 0 [IQR: 0.0-3.3]. Uveitis-complicated JIA was documented in 6 (5.9%) patients. Regarding medications, 39 (38.6%) patients were on non-steroidal anti-inflammatory drugs (NSAIDs), and 30 (29.7%) patients were on systemic corticosteroids. Methotrexate was prescribed in 54 (53.5%) patients. Seven patients were treated with biologic agents, including tocilizumab in 4 patients, etanercept in 2 patients, and infliximab in 1 patient. Demographic and clinical characteristics is shown in the Table 1.

**Table 1:** Demographic and clinical characteristics of children and adolescents with JIA (N = 101)

Characteristics	Values		
Male, n (%)	50 (49.5)		
Subtypes			
Systemic	26 (25.7)		
Polyarticular, RF-	9 (8.9)		
Polyarticular, RF+	12 (11.9)		
Oligoarthritis	14 (13.9)		
Enthesitis-related arthritis	34 (33.7)		
Psoriatic arthritis	0 (0)		
Undifferentiated	6 (5.9)		
Age at study visit (years), median [IQR]	11.8 [8.4–14.3]		
Age at disease onset (years), median [IQR]	7.9 [3.8–9.9]		
Disease duration at time of assessment (months), median [IQR] Duration from symptoms onset to time of assessment (months), median [IQR]	32.7 [11.7–58.4] 39.6 [17.9–65.4]		
Duration from symptoms onset to diagnosis (months), median [IQR]	3.0 [1.2–8.2]		
Body mass index (kg/m^2^), median [IQR] Family history of JIA	18.9 [16.0-21.6] 2 (1.9)		
Health care coverage scheme			
Universal coverage Civil servant medical benefit Self-payment	77 (76.2) 12 (11.9) 12 (11.9)		
Disease activity			
Active disease, n (%)	35 (34.7%)		
Inactive disease, n (%)	34 (33.7%)		
Remission on medication, n (%)	4 (4.0%)		
Remission off medication, n (%)	28 (27.7%)		
CHAQ score, median [IQR]	0.0 [0.0-0.3]		
NSAIDs, n (%)	39 (38.6)		
Systemic corticosteroids, n (%) Intra-articular corticosteroids injection, n (%)	30 (29.7) 54 (53.5)		
DMARDs, n (%)			
Methotrexate	54 (53.5)		
Sulfasalazine	14 (13.9)		
Cyclosporin-A	3 (3.0)		
Biologics, n (%)			
Tocilizumab	4 (4.0)		
Etanercept	2 (2.0)		
Infliximab	1 (1.0)		

Abbreviations: CHAQ, Child health assessment questionnaire; DMARDs, disease-modifying antirheumatic drugs; IQR, interquartile range; JIA, juvenile idiopathic arthritis; NSAIDs, non-steroidal anti-inflammatory drugs; RF, rheumatoid factor

Moderate to severe disability was found in 20 (19.8%) patients. Using the Steinbrocker functional classification system, 83 (82.2%) patients were class I, 15 (14.9%) patients were class II, 0 (0.0%) patients were class III, and 3 (3.0%) patients were class IV.

Thirty-seven of 101 (36.6%) patients had articular damage (JADI-A ≥ 1). The elbow joint limitation was the most common type of articular damage (12 patients, 11.9%), followed by lumbar spine restriction (11 patients, 10.9%) and hip damage (9 patients, 8.9%). The distribution of joint damage compared among the JIA subtypes is shown in Table 2.

**Table 2:** JADI-A and mJADI-A evaluation items compared among the JIA subtypes

Sites	Total (N = 101) n (%)	SJIA (n = 26) n (%)	Poly RF- (n = 9) n (%)	Poly RF+ (n = 12) n (%)	Oligo (n = 14) n (%)	ERA (n = 34) n (%)	Undifferentiated (n = 6) n (%)
TMJ	2 (2.0)	1 (3.8)	-	-	-	1 (2.9)	-
C-spine	2 (2.0)	2 (7.7)	-	-	-	-	-
L-spine	11 (10.9)	-	-	-	-	10 (29.4)	1 (16.7)
Shoulder	2 (2.0)	2 (7.7)	-	-	-	-	-
Elbow	12 (11.9)	4 (15.4)	2 (22.2)	2 (16.7)	1 (7.1)	3 (8.8)	-
Wrist	9 (11.0)	4 (17.4)	-	2 (20.0)	2 (15.4)	1 (3.8)	-
MCP	2 (2.0)	1 (3.8)	-	1 (8.3)	-	-	-
PIP	6 (5.9)	2 (7.7)	3 (33.3)	-	-	1 (2.9)	-
Hip	9 (8.9)	4 (15.4)	-	-	-	5 (14.7)	-
Knee	6 (5.9)	2 (7.7)	-	1 (8.3)	1 (7.1)	2 (5.9)	-
Ankle	6 (5.9)	2 (7.7)	-	2 (16.7)	-	2 (5.9)	-
Tarsal	4 (4.0)	1 (3.8)	-	-	-	3 (8.8)	-
MTP	1 (1.0)	1 (3.8)	-	-	-	-	-

Abbreviations: ERA, enthesitis-related arthritis; JADI-A, Juvenile Arthritis Damage Index Articular; mJADI-A, modified JADI-A; JIA, juvenile idiopathic arthritis; L-spine, lumbar spine; MCP, metacarpophalangeal joint; MTP, metatarsophalangeal joint; PIP, proximal interphalangeal joints; RF, rheumatoid factor; SJIA, systemic juvenile idiopathic arthritis; TMJ, temporomandibular joint

Extra-articular damage was observed in 25 (24.8%) patients. Growth failure and striae were similarly predominant complications in 8 (7.9%) patients. Leg-length discrepancy and muscle atrophy were documented in 5 (5%) patients. Avascular necrosis, scoliosis, osteoporosis with fracture, delayed puberty, diabetes mellitus, aortitis, and ocular damage were found in 1 (1.0%) patient each (Table 3).

**Table 3:** JADI-E and mJADI-E evaluation items compared among the JIA subtypes

Items	Total (N = 101) n (%)	SJIA (n = 26) n (%)	Poly RF- (n = 9) n (%)	Poly RF+ (n = 12) n (%)	Oligo (n = 14) n (%)	ERA (n = 34) n (%)	Undifferentiated (n = 6) n (%)
Ocular	1 (1.0)	-	-	-	-	1 (2.9)	-
MSK atrophy	5 (5.0)	2 (7.7)	-	-	-	3 (8.8)	-
Osteoporosis with fracture	1 (1.0)	1 (3.8)	-	-	-	-	-
AVN	1 (1.0)	1 (3.8)	-	-	-	-	-
Scoliosis	1 (1.0)	1 (3.8)	-	-	-	-	-
LLD	5 (5.0)	2 (7.7)	-	-	1 (7.1)	2 (5.9)	-
Striae	8 (7.9)	3 (11.5)	-	-	1 (7.1)	4 (11.8)	-
Post TA IA atrophy	5 (5.0)	2 (7.7)	-	-	2 (14.3)	1 (2.9)	-
Growth failure	8 (7.9)	6 (23.1)	-	-	-	1 (2.9)	1 (16.7)
Delayed puberty	1 (1.0)	1 (3.8)	-	-	-	-	-
DM with treatment	1 (1.0)	-	1 (11.1)	-	-	-	-
Amyloidosis	-	-	-	-	-	-	-
Aortitis	1 (1.0)	-	-	-	-	1 (2.9)	-

Abbreviations: AVN, avascular necrosis; DM, diabetes mellitus; ERA, enthesitis-related arthritis; IA, intra-articular injection; JADI-E, Juvenile Arthritis Damage Index Extra-articular (JADI-E); mJADI-E, modified JADI-E; JIA, juvenile idiopathic arthritis; LLD, leg-length discrepancy; MSK, musculoskeletal; RF, rheumatoid factor; SJIA, systemic juvenile idiopathic arthritis; TA, triamcinolone acetonide

Concerning comorbidities, 15 (14.9%) patients had infection. The most commonly observed infections were upper respiratory tract infection (5 patients), pneumonia (2 patients), bronchitis (4 patients), and gastroenteritis (3 patients). Varicella and herpes zoster infection were each found in one patient only. No JIA patients had tuberculosis. Hypertension developed in 6 patients, and glaucoma was detected in 4 patients. Nonalcoholic steatohepatitis was detected in 2 patients. Macrophage activation syndrome was complicated in 5 (19.2%) patients with systemic JIA. No malignancy was observed in this study.

Multivariable logistic regression analysis revealed Steinbrocker functional classification greater than class I (adjusted odds ratio [aOR]: 18.1, 95% confidence interval [CI]: 3.9–84.6; *p* < 0.001), delayed diagnosis ≥ 6 months (aOR: 8.5, 95%CI: 2.7–27.0; *p* < 0.001), and ERA subtype (aOR: 5.7, 95%CI: 1.8–18.3; *p* = 0.004) to be significantly and independently associated with articular damage in Thai children and adolescents with JIA (Table 4). Systemic corticosteroids treatment was the independent factor associated with extra-articular damage with the aOR of 3.8 (95% CI: 1.3–11.1, *p* = 0.013) (Table 5).

**Table 4:** Factors associated with articular damage in JIA

Factors	Univariable logistic regression			Multivariable logistic regression		
	**Crude OR**	**(95%CI)**	***p***	**Adjusted** **OR**	**(95%CI)**	***p***
Enthesitis-related arthritis	4.2	(1.7–10.1)	0.001*	5.7	(1.8–18.3)	0.004*
Systemic corticosteroids use	1.5	(0.6–3.6)	0.365			
Intra-articular corticosteroids use	2.5	(1.1–5.9)	0.033*	2.7	(0.9–8.4)	*0.081*
DMARDs use	2.0	(0.8–4.6)	0.125			
Biologics use	2.5	(0.5–11.7)	0.256			
Severe CHAQ	3.6	(0.3–41.1)	0.303			
Steinbrocker > class I	13.9	(3.7–52.5)	< 0.001*	18.1	(3.9–84.6)	< 0.001*
Delayed diagnosis ≥ 6 months	5.7	(2.3–14.0)	< 0.001*	8.5	(2.7–27.0)	< 0.001*
Disease duration ≥ 5 years	1.3	(0.5–3.4)	0.558			

*A *p-*value < 0.05 indicates statistical significance

Abbreviations: CHAQ, Child Health Assessment Questionnaire; CI, confidence interval; DMARDs, disease-modifying antirheumatic drugs; OR, odds ratio

**Table 5:** Factors associated with extra-articular damage in JIA

Factors	Univariable logistic regression			Multivariable logistic regression		
	**Crude OR**	**(95%CI)**	***p***	**Adjusted** **OR**	**(95%CI)**	***p***
Enthesitis-related arthritis	1.1	(0.4–2.9)	*0.776*			
Systemic corticosteroids use	4.8	(1.8–12.5)	0.001*	3.8	(1.3–11.1)	0.013*
Intra-articular corticosteroids use	0.7	(0.3–1.8)	*0.528*			
DMARDs use	1.2	(0.5–3.1)	*0.671*			
Biologics use	9.3	(1.7–51.2)	0.011*	4.4	(0.6–31.0)	*0.140*
Severe CHAQ	6.5	(0.6–75.2)	*0.133*			
Delayed diagnosis ≥ 6 months	0.7	(0.3-2.0)	*0.566*			
Disease duration ≥ 5 years	1.8	(0.6–4.8)	*0.268*			
Obesity (BMI ≥ 25 kg/m^2^)	4.3	(1.4–13.0)	0.011*	2.1	(0.6–7.6)	0.260

*A *p-*value < 0.05 indicates statistical significance

Abbreviations: BMI; Body Mass Index; CHAQ, Child health assessment questionnaire; CI, Confidence interval; DMARDs, Disease-modifying antirheumatic drugs; JIA, Juvenile idiopathic arthritis; OR, Odds ratio

## Discussion

Our study demonstrates that disability was recognized in one-fifth of the Thai children and adolescents with JIA and one-third of the patients were found to have disease-related damage. We found that ERA subtype and delayed diagnosis were significantly associated with articular damage.

During the biologic era, disability in patients with JIA has decreased. A study found that patients with systemic JIA and patients with polyarticular JIA who were treated with tocilizumab had significantly reduced disability [29]. Tanya, et al. reported disability and joint damage to be rare in their Singaporean JIA cohort; and 36% of their patients were treated with biologic agents [30]. In the present study, almost 20% of patients were categorized as moderate to severe disability and only 6.9% of our patients received biologic agents. The treatment with biologic agents was substantially lower at our centre attributed to the limited access to biologic agents in resource-restricted countries [6]. Therefore, improved access to biologic agents, for treating JIA is necessary [31].

Of our 101 included Thai JIA patients, 37 (36.6%) had articular damage. This finding is consistent with the findings of other Asian studies that reported a prevalence of articular damage ranging from 27.5 to 60.7% [7, 9, 12, 21]. Sarma, et al. reported articular damage in 60.7% of JIA patients in India [7]. This high rate of disease-related damage could be secondary to the long median disease duration of 60 months [IQR: 12–240] [7]. In contrast, Rypdal, et al. reported a lower rate of articular damage (13.4%) in their Nordic JIA patient cohort [11]. Importantly, Giancane, et al. reported JIA disease-related damage to be decreased during the biologic era when compared to the rate of damage observed during the methotrexate era (11% oligoarthritis and 21.8% polyarthritis during the biologic era vs. 17.6% oligoarthritis and 52.6% polyarthritis during the methotrexate era) [32]. Articular damage was found in only 5% of Greek JIA patients, and 40.8% of the patients in that study received biologic agents to treat their JIA [10]. These results describe the outcomes of patients in more developed countries, but they may not reflect the outcomes of JIA patients in resource-restricted countries.

Different subtypes of JIA influence different types of disease-related damage. Multivariable regression analysis in our study revealed ERA to be an independent predictor of articular damage, and lumbar spine and the hip were the two most commonly damaged joints in ERA. Hip involvement was previously reported to be one of the predictors of poor outcome in ERA through adulthood [33]. Moreover, ERA was found to have the highest JADI-A score in an Arab cohort [12]. Up to 34.7% of ERA patients had articular damage, and the hip was the most commonly damaged joint [7]. In contrast, the polyarticular JIA subtype was found to be a predictor of damage reported by Sarma et al. and Rypdal et al.; however, those two studies had a lower proportion of ERA subtype than we had in the present study [7, 11]. Our data showed the ERA subtype to be the predominant JIA subtype, which is similar to the results from other JIA cohorts in Asian countries, but dissimilar from JIA cohorts from Western countries that were studied [30, 34, 35]. Al-Mayouf et al. interestingly reported that articular damage was associated with the presence of a family history of JIA [12]. These suggest the likelihood of genetic factors that influence the subtypes of JIA. The median duration from symptoms onset to diagnosis was 3.8 [1.7–7.3] months in ERA patients and 2.8 [1.0-9.4] months for non-ERA patients, with no statistically significant difference (*p*-value = 0.354). Therefore, the finding that ERA as a predictor of articular damage was less likely interfered by the time to diagnosis.

Our results also revealed delayed diagnosis ≥ 6 months to be independently associated with articular damage. Regarding the referral system in Thailand, paediatric rheumatologists receive referral of children suspected of rheumatic diseases from several pathways such as from general paediatricians, general practitioners, orthopaedic surgeons and adult rheumatologists. Patients can also be seen by paediatric rheumatologists directly if their caregivers recognize the symptoms concerning the development of rheumatic diseases. Based on our previous study, in 285 patients with musculoskeletal (MSK) complaints who were referred to our service, they were referred by general paediatricians at 33.3% and general practitioners at 10.5% [36]. Early referral to receive appropriate care in patients with JIA could minimize damage [34]. The recommendations regarding JIA management in less resourced countries (JAMLess) suggest that the patients whom suspected JIA should be evaluated by a paediatric rheumatologist within 4 weeks in order to improve outcomes [5]. Notably, 32.7% of patients in our study had delayed diagnosis ≥ 6 months. Therefore, raising social awareness about JIA, enhancing education about paediatric rheumatic diseases for clinicians, and improving access to care are needed.

Extra-articular damage was observed in 24.8% of our JIA patients, and growth failure was the main damage observed. Previous study reported that inflammatory cytokines, including interleukin (IL)-6, IL-1β, and anti-tumour necrosis factor alpha have a negative impact on growth by altering both growth hormone and insulin-like growth factor-1 [37]. Another possible factor is that 29.7% of our patients were on systemic corticosteroids. Our study also pointed out that systemic corticosteroids use was the independent predictor of extra-articular damage. It is well-recognized that chronic corticosteroids exposure disrupts growth hormone release and impairs insulin-like growth factor-1 signalling, and the subsequent result is growth failure [37]. Growth failure was found in 20-28.7% in previous studies [8, 12]. These results clearly indicate that growth failure is an important complication in children and adolescents with JIA. Accordingly, growth assessment and monitoring with prompt intervention should be component of the patient care protocol in children and adolescents with JIA.

Our study found uveitis-complicated JIA in only 6 (5.9%) patients. This finding was consistent with other studies in Asians that reported a low prevalence of uveitis-complicated JIA [30, 38–40]. Prevalence of JIA-associated uveitis was detected at 11.6% of African American and Non-Hispanic White children in the Childhood Arthritis and Rheumatology Research Alliance Registry [41]. Uveitis was found in 15–19% in 3 JIA registries from the United Kingdom [42]. Based on the Nordic cohort, uveitis developed in 22.1% of JIA patients [43]. The observed lower prevalence of uveitis-complicated JIA could be secondary to the lower proportion of oligoarticular JIA subtype in Asians [34]. Even though the prevalence of JIA-associated varied, all patients with JIA should undergo uveitis surveillance and receive appropriate treatment [26, 44]. Comparison of disease-related damage in children and adolescents with JIA among different studies is shown in Table 6.

**Table 6:** Studies of disease-related damages in children and adolescents with JIA

Year, Authors	Countries/Groups	Patients, N	JIA subtypes, n (%)							Disease duration, median [IQR]	JADI-A, n (%)	JADI-E, n (%)	Associated factor for articular damage	Associated factor for extra-articular damage
			SJIA	PJIARF-	PJIARF+	OJIA	ERA	PsA	UJIA					
This study	Thailand	101	26 (25.7)	9 (8.9)	12 (11.9)	14 (13.9)	34 (33.7)	-	6 (5.9)	32.7 [11.7–58.4] months	37 (36.6)	25 (24.8)	- Delayed diagnosis ≥ 6 months - ERA subtype - SFC > class I	- Systemic corticosteroids use
2021, Al-Mayouf et al. [12]	PRAG group	702	172 (24.5)	159 (22.6)	48 (6.8)	245 (34.9)	39 (5.6)	28 (4)	11 (1.6)	48 [24–84] months	193 (27.5)	156 (22.2)	- Family history of JIA - Disease duration - Delayed treatment of DMARDs, biologics - Hip involvement - Wrist involvement	- Disease duration - Delayed treatment of DMARDs, biologics - Hip involvement - Wrist involvement
2018, Menon et al. [9]	India	62	20 (32.3)	24 (38.7)	2 (3.2)	15 (24.2)	1 (1.6)	-	-	24 [7-60.8] months	19 (30.6)	15 (24.2)	- Disease duration - Younger age of onset	- Disease duration
2018, Nalbanti et al. [10]	Greece	120	8 (6.7)	33 (27.5)	-	69 (57.5)	4 (3.3)	6 (5)	-	8 [4–14.1] years	6 (5)	9 (7.5)	-Cumulative time of active disease	- Cumulative time of active disease
2018, Rypdal et al. [11]	Nordic cohort	423	-	94 (22.2)	4 (1)	227 (53.7)	34 (8)	6 (1.4)	58 (13.7)	98 [95–102] months	29 (13.4)	N/A	- Age at disease onset - Number of active joint count - PGA-VAS - CHAQ	N/A
2012, Singh et al. [24]	India	101	-	-	-	-	101 (100)	-	-	6 [1–24] years	58 (57.4)	35 (34.7)	N/A	N/A
2011, Susic et al. [8]	Serbia	87	13 (14.9)	22 (25.3)	10 (11.5)	31 (35.6)	11 (12.6)	-	-	5.2 [3.5–8.5] years	36 (41.4)	34 (39.1)	- Number of limited joints motion - Cumulative duration of active disease - Functional class - Radiological damage - JADI-E	N/A
2008, Sarma et al. [7]	India	89	23 (25.8)	35 (39.3)	12 (13.5)	15 (16.8)	-	1 (1.1)	3 (3.4)	5 [1–20] years	54 (60.7)	35 (39.3)	- PGA, PGW - Radiological damage - CHAQ - JADI-E - Disease duration - ESR - Morning stiffness - Loss of education - Number of active joint count	N/A
2008, Solari et al. [21]	Italy	310	31 (10)	65 (21)	5 (1.6)	168 (54.2)	-	17 (5.5)	24 (7.7)	6.7 [5.4–9.3] years	101 (32.4)	81 (26.1)	N/A	N/A

Abbreviations: CHAQ, Child health assessment questionnaire; DMARDs, Disease-modifying antirheumatic drugs; ERA, Enthesitis-related arthritis; ESR, Erythrocyte sedimentation rate; JADI-A, Juvenile arthritis damage index articular; JADI-E, Juvenile arthritis damage index extra-articular; JIA, Juvenile idiopathic arthritis; MTX, Methotrexate; N/A, not available; OJIA, Oligoarticular JIA; PJIA, Polyarticular JIA; PsA, Psoriatic arthritis; RF, Rheumatoid factor; SFC, Steinbrocker functional classification; SJIA, Systemic JIA; PGA, Physician global assessment of overall disease activity; PGW, Patient’s or parent’s assessment of overall well-being; UJIA, Undifferentiated JIA; VAS, Visual analogue scale

This study also has some mentionable limitations. First, this study enrolled a relatively small number of patients from a single tertiary care centre, although our centre is the largest national tertiary referral centre in Thailand. Second, the cross-sectional design of our study and the relatively short follow-up period render it unable to reliably predict long-term damage and disability which should be interpreted with caution. However, the patients in our study had a median disease duration of 32.7 months, and disease-related damage was apparent in up to one-third of patients. It can be argued that the results of our study reflect the real-world outcomes of paediatric JIA in a resource-restricted country during the biologic era. Third, due to cost- and waiting list-related challenges, we were unable to obtain magnetic resonance imaging to evaluate for subtle sacroiliitis, which could affect JADI scoring. However, we included JADI and m-JADI combined with retrospective comorbidity data collection in order to more comprehensively assess the damage and complications in all subtypes of JIA, including ERA.

Our study added important messages to general paediatricians and/or general practitioners to early detect children with chronic arthritis suspected of JIA with prompt referral to paediatric rheumatologists. More MSK education should be enhanced during the training in both undergraduate and postgraduate levels. Additionally, public awareness of JIA needs to be endorsed. In terms of the evaluation and follow up of patients with JIA by paediatric rheumatologists, patients with ERA should be more focused as this subtype was found to be the independent predictor of articular damage. Moreover, not only appropriate aggressive treatment according to the treat to target recommendations [2], physiotherapy and occupational therapy should be a part of multidisciplinary team at the beginning of JIA management. Physiotherapists and occupational therapists specialized in JIA should be promoted in the resource-restricted countries including Thailand.

## Conclusion

Disability and disease-related damage was identified in one-fifth and one-third of Thai patients with JIA, respectively. Steinbrocker functional classification greater than class I, delayed diagnosis ≥ 6 months, and ERA could predict articular damage. Systemic corticosteroids use was the predictor of extra-articular damage. Early detection, diagnosis, and appropriate treatment are essential for preventing or reducing permanent damage and disability in children and adolescents with JIA.

## Data Availability

The datasets used and/or analysed during the current study are available upon reasonable request.

## Abbreviations

aOR: Adjusted odds ratio
BMI: Body mass index
CHAQ: Child health assessment questionnaire
CI: Confidence interval
CRP: C-reactive protein
ESR: Erythrocyte sedimentation rate
ERA: Enthesitis-related arthritis
IL: Interleukin
JADAS: Juvenile arthritis disease activity score
JADI: Juvenile arthritis damage index
JADI-A: Juvenile arthritis damage index-articular
JADI-E: Juvenile arthritis damage index-extra-articular
JAMLess: Juvenile arthritis management in less resourced countries
JIA: Juvenile idiopathic arthritis
mJADI: Modified-juvenile arthritis damage index
mJADI-A: Modified-juvenile arthritis damage index-articular
mJADI-E: Modified-juvenile arthritis damage index-extra-articular
MSK: Musculoskeletal
NSAIDs: Non-steroidal anti-inflammatory drugs
PGA: Physician global assessment of overall disease activity
PGW: Parent’s or patient’s assessment of overall well-being
RF: Rheumatoid factor
STROBE: Strengthening the reporting of observational studies in epidemiology
